# Comparative chloroplast genomics reveals the phylogeny and the adaptive evolution of *Begonia* in China

**DOI:** 10.1186/s12864-023-09563-3

**Published:** 2023-10-27

**Authors:** Chao Xiong, Yang Huang, Zhenglong Li, Lan Wu, Zhiguo Liu, Wenjun Zhu, Jianhui Li, Ran Xu, Xin Hong

**Affiliations:** 1 School of Life Science and Technology, Wuhan Polytechnic University, Wuhan, Hubei, 430023 People’s Republic of China; 2 Anhui Provincial Engineering Laboratory of Wetland Ecosystem Protection and Restoration, School of Resources and Environmental Engineering, Anhui University, Hefei, Anhui, 230601 People’s Republic of China; 3 College of Tourism and Landscape Architecture, Guilin University of Technology, Guilin, Guangxi, 541006 People’s Republic of China; 4 Institute of Chinese Materia Medica, China Academy of Chinese Medical Sciences, Beijing, 100700 People’s Republic of China; 5 College of Chemistry and Material Engineering, Quzhou University, Quzhou, Zhejiang 324000 People’s Republic of China

**Keywords:** *Begonia*, Chloroplast, Phylogeny, Evolutionary, Positive selection

## Abstract

**Background:**

The *Begonia* species are common shade plants that are mostly found in southwest China. They have not been well studied despite their medicinal and decorative uses because gene penetration, decreased adaptability, and restricted availability are all caused by frequent interspecific hybridization.

**Result:**

To understand the patterns of mutation in the chloroplast genomes of different species of *Begonia*, as well as their evolutionary relationships, we collected seven *Begonia* species in China and sequenced their chloroplast genomes. *Begonia* species exhibit a quadripartite structure of chloroplast genomes (157,634 − 169,694 bp), consisting of two pairs of inverted repeats (IR: 26,529 − 37,674 bp), a large single copy (LSC: 75,477 − 86,500 bp), and a small single copy (SSC: 17,861 − 18,367 bp). 128–143 genes (comprising 82–93 protein-coding genes, 8 ribosomal RNAs, and 36–43 transfer RNAs) are found in the chloroplast genomes. Based on comparative analyses, this taxon has a relatively similar genome structure. A total of six substantially divergent DNA regions (*trn*T-UGU-*trn*L-UAA, *atp*F-*atp*H, *ycf*4-*cem*A, *psb*C-*trn*S-UGA, *rpl*32-*trn*L-UAG, and *ccs*A-*ndh*D) are found in the seventeen chloroplast genomes. These regions are suitable for species identification and phylogeographic analysis. Phylogenetic analysis shows that *Begonia* species that were suited to comparable environments grouped in a small clade and that all *Begonia* species formed one big clade in the phylogenetic tree, supporting the genus’ monophyly. In addition, positive selection sites were discovered in eight genes (*rpo*C1, *rpo*B, *psb*E, *psb*K, *pet*A, *rps*12, *rpl*2, and *rpl*22), the majority of which are involved in protein production and photosynthesis.

**Conclusion:**

Using these genome resources, we can resolve deep-level phylogenetic relationships between *Begonia* species and their families, leading to a better understanding of evolutionary processes. In addition to enhancing species identification and phylogenetic resolution, these results demonstrate the utility of complete chloroplast genomes in phylogenetically and taxonomically challenging plant groupings.

**Supplementary Information:**

The online version contains supplementary material available at 10.1186/s12864-023-09563-3.

## Background

Sunlight is essential for the growth of most heliophilous plants, and in severe cases, they may even die due to a lack of light. There are, however, a variety of *Begonia* species that can adapt to a range of light levels and have a wide range of forms, they are widely distributed and attached to caves in humid and shady environments [1]. Listed among the top ten large angiosperm genera, *Begonia* is a member of the Begoniaceae family with over 2,000 described species [2]). In tropical and subtropical regions, *Begonia* diversity varies unevenly. American and Asian species are the most diverse, with each having over 600 species, while African species are relatively scarce with only 160 species, and Australian species are absent [3]. A total of 130 *Begonia* species are included in the Flora of China, which occurs naturally in the south of the Yangtze River basin. Because of its wide variety of morphological characteristics, *Begonia* is an excellent species for studying shade-adapted plants in China. Particularly distinctive are its asymmetrical leaves, monoecious blooms, and three-winged capsules, which are dry and papillose [4]. *Begonia* serves as a fantastic system for examining the processes and patterns underlying the production of biodiversity because it is a megadiverse, pantropically distributed genus [5]. Analyzing plastid genome (plastome) structure and repeat content was necessary in order to gain insight into how the plastid genome might play a role in species evolution.

As a means of addressing adaptive evolution in plants, the plastomes can be useful. Under different light intensities or different living environments, some genes will leave fingerprints in plastomes. These genes are associated with photosynthesis and genetic systems, which play an important role in helping plants adapt to various environments [6, 7]. In addition, comparative genomic analysis of chloroplasts can be used to identify areas of high variability and create specific molecular markers of populations or species for use in species identification. With the decrease in sequencing costs and the continuous improvement of sequencing technology in recent years, more and more plant plastomes have been successfully sequenced and applied to species identification and phylogenetic evolution studies. However, few chloroplast genomes have been published in *Begonia*, most of which have focused on biodiversity studies of species using DNA barcode fragments. To resolve section-level patterns of phylogenetic diversity in south American *Begonia*, for instance, Moonlight et al. [8] used three plastid regions (the *ndh*A intron, the *ndh*F-*rpl*32 spacer, and the *rpl*32-*trn*L spacer), but there was no robust resolution at the species level, which restricts the analysis of the adaptability of *Begonia* to the geographical environment. In order to comprehend the development of the chloroplast genomes and reconstruct the phylogenetic connections of *Begonia*, a comparative study of the chloroplast genome is of great utility.

In this study, we focus on the mechanisms of diversity formation and adaptive evolution in *Begonia*, which will be conducive to the study of evolution at different levels, from population to species to the whole genus. Here we present chloroplast genome information for seven different species of *Begonia*, which will serve as the foundation for a new reference sequence database and be used to map the genetic structure of the genus. These genetic resources provide tools for discovering the functional genetic basis of population variation in *Begonia* species and mapping adaptation analyses at the interspecific and population levels.

## Results

### Plastid genome features

**Fig. 1:**
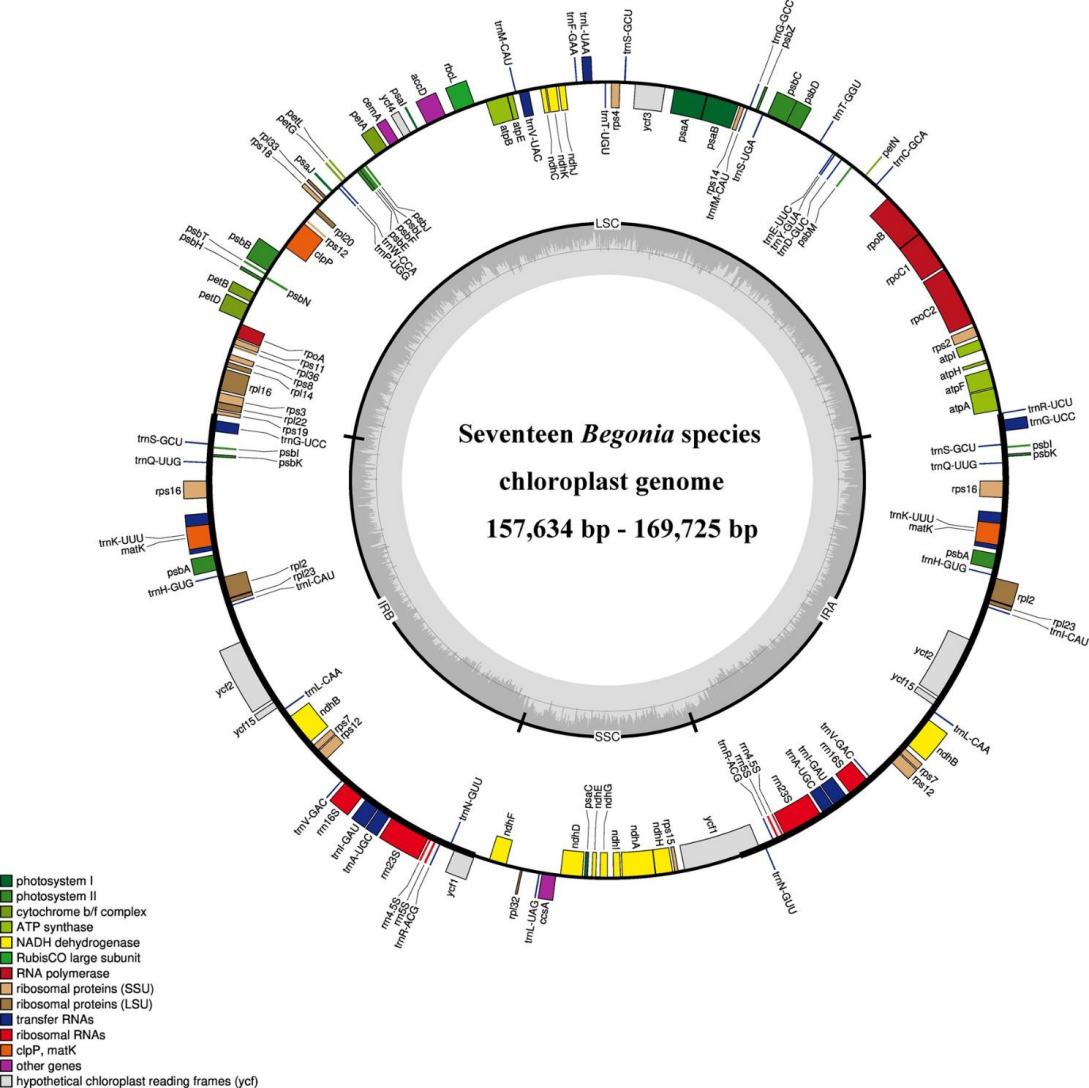
Structural map of the *Begonia* chloroplast genome. Genes shown outside the outer circle are transcribed clockwise and those inside are transcribed counterclockwise. Genes belonging to different functional groups are color-coded. *B. cathayana* is used as the template for Fig. 1. The dark grey plot in the inner circle corresponds to GC content. Large single copy, small single copy, and inverted repeat are indicated with LSC, SSC, and IR (IRa and IRb), respectively

In this study, a total of seven *Begonia* plants were collected, and their chloroplast genomes were sequenced and assembled. We discovered that all 17 *Begonia* cp. genomes (the other 10 cp. genome sequences are available from NCBI) have a circular DNA molecule and a typical quadripartite structure, which includes a small single copy region (SSC), a large single copy region (LSC), and two inverted repeat regions (IRa and IRb) (Fig. 1). Among all 17 *Begonia* species, the complete chloroplast genomes ranged from 157,634 bp (*B. guangxiensis*) to 169,694 bp (*B. smithiana*) in length (Table 1), and the length of the LSC, SSC, and IR regions is from 75,477 bp (*B. cavaleriei*) to 86,500 bp (*B. guangxiensis*), from 17,861 bp (*B. emeiensis*) to 18,367 bp (*B. cathayana*), from 26,529 bp (*B. guangxiensis*) to 37,674 bp (*B. versicolor*), respectively. The cp. genomes of *Begonia* have similar GC levels, *B. leprosa* had the lowest GC content (35.47%) and *B. obsolescens* had the greatest GC content (35.90%).

**Table 1:** Summary of the chloroplast genomes of seventeen *Begonia* species

Species	Genome Length (bp)	LSC Length (bp)	SSC Length (bp)	IR Length (bp)	GC (%)	Total Genes	CDS	tRNA	rRNA
*B. cathayana* ◆	169,164	75,611	18,367	37,593	35.58	137	88	41	8
*B. cavaleriei* ◆	168,857	75,477	18,264	37,558	35.53	139	89	42	8
*B. grandis* ◆	169,050	76,155	18,141	37,377	35.66	139	89	42	8
*B. leprosa* ◆	168,908	75,647	18,135	34,145	35.47	137	88	41	8
*B. obsolescens* ◆	167,409	81,016	18,103	34,145	35.90	133	87	38	8
*B. smithiana* ◆	169,694	76,112	18,310	37,636	35.59	137	88	41	8
*B. umbraculifolia* ◆	169,682	76,454	18,088	37,570	35.49	138	88	42	8
*B. arachnoidea*	169,725	76,431	18,146	37,574	35.50	140	90	42	8
*B. asteropyrifolia*	169,512	76,310	18,058	37,572	35.50	139	89	41	8
*B. coptidifolia*	169,412	75,938	18,262	37,606	35.57	130	82	40	8
*B. emeiensis*	169,079	76,006	17,861	37,606	35.60	138	88	42	8
*B. ferox*	169,114	75,887	18,105	37,561	35.49	143	93	42	8
*B. guangxiensis*	157,634	86,500	18,076	26,529	35.89	128	84	36	8
*B. gulongshanensis*	169,153	75,998	18,063	37,546	35.52	140	90	42	8
*B. handelii*	169,406	75,901	18,315	37,595	35.56	139	89	42	8
*B. pulchrifolia*	169,589	76,057	18,320	37,606	35.56	142	91	43	8
*B. versicolor*	169,506	75,868	18,290	37,674	35.57	138	89	41	8

◆— newly sequenced species

A total of 128–142 genes were identified based on gene annotation, of which comprised 82–93 protein-coding genes, 36–43 transfer RNAs (tRNAs), and 8 ribosomal RNAs (rRNAs) (Table 1), whereas the number of genes fluctuates between species as a result of IRs contraction and expansion. These genes were separated into three groups: 59 genes are related to self-replication (the large subunit of the ribosome, the small subunit of the ribosome, and RNA polymerase), 43 genes are involved in photosynthesis (photosystem I, photosystem II, cytochrome b/f complex, ATP synthase, Rubisco large subunit, and NADPH dehydrogenase), and other genes are associated with related enzymes (ATP-dependent protease, Maturase, Acetyl-CoA carboxylase, Cytochrome c biogenesis, and Inner membrane protein) (Table 2).

**Table 2:** Genes in the chloroplast genome of seventeen *Begonia* species

Category	Gene group	Gene name
Protein synthesis and DNA-replication	Ribosomal RNA genes	*rrn4.5#, rrn5#, rrn16#, rrn23#*
	Transfer RNA genes	trnA-UGC^*^#, trnC-GCA, trnD-GUC, trnE-UUC, trnF-GAA, trnG-GCC, trnG-UCC*#, trnH-GUG#, trnI*, trnI-CAU#, trnI-GAU^*^#, trnK-UUU^*^#, trnL-CAA#, trnL-UAA^*^, trnL-UAG, trnM-CAU, trnN-GUU#, trnP-UGG, trnQ-UUG#, trnR-UCU, trnR-ACG#, trnS-GCU, trnS-UGA, trnT-GGU, trnT-UGU, trnV-GAC#, trnV-UAC^*^, *trnW-CCA, trnY-GUA, trnfM-CAU*
	Ribosomal protein genes (larger subunit)	rpl2^*^#, rpl14, rpl16^*^, *rpl20, rpl22, rpl23#, rpl32, rpl33, rpl36*
	Ribosomal protein genes (smaller subunit)	rps2, rps3, rps4, rps7#, rps8, rps11, rps12**#, rps14, rps15, rps16*#, rps18, rps19
	RNA polymerase	rpoA, rpoB, rpoC1^*^, *rpoC2*
Photosynthesis	Photosystem I	*psaA, psaB, psaC, psaI, psaJ*
	Photosystem II	*psbA#, psbB, psbC, psbD, psbE, psbF, psbH, psbI#, psbJ, psbK#, psbL, psbM, psbN, psbT*
	Cytochrome b/f complex	petA, petB, petD*#, petG, petL, petN
	ATP synthase	atpA, atpB, atpE, atpF^*^, *atpH, atpI*
	Rubisco large subunit	*rbcL*
	NADH dehydrogenase	ndhA^*^, ndhB^*^#, ndhC, ndhD, ndhE, ndhF^*^, *ndhG, ndhH, ndhI, ndhJ, ndhK*
Miscellaneous group	ATP-dependent protease	*clpP* ^**^
	Maturase	*matK#*
	Acetyl-CoA carboxylase	*accD*
	Cytochrome c biogenesis	*ccsA*
	Inner membrane protein	*cemA*
	Translational initiation factor	*infA*
	Ribosomal protein L16	*rplP*
Pseudogene unknown function	Hypothetical chloroplast reading frames (*ycf*)	ycf1*#, ycf2#, ycf3^**^, *ycf4, ycf15#*

*—Gene containing a single intron; **—Gene containing two introns; #—Multi-copy genes

**Fig. 2:**
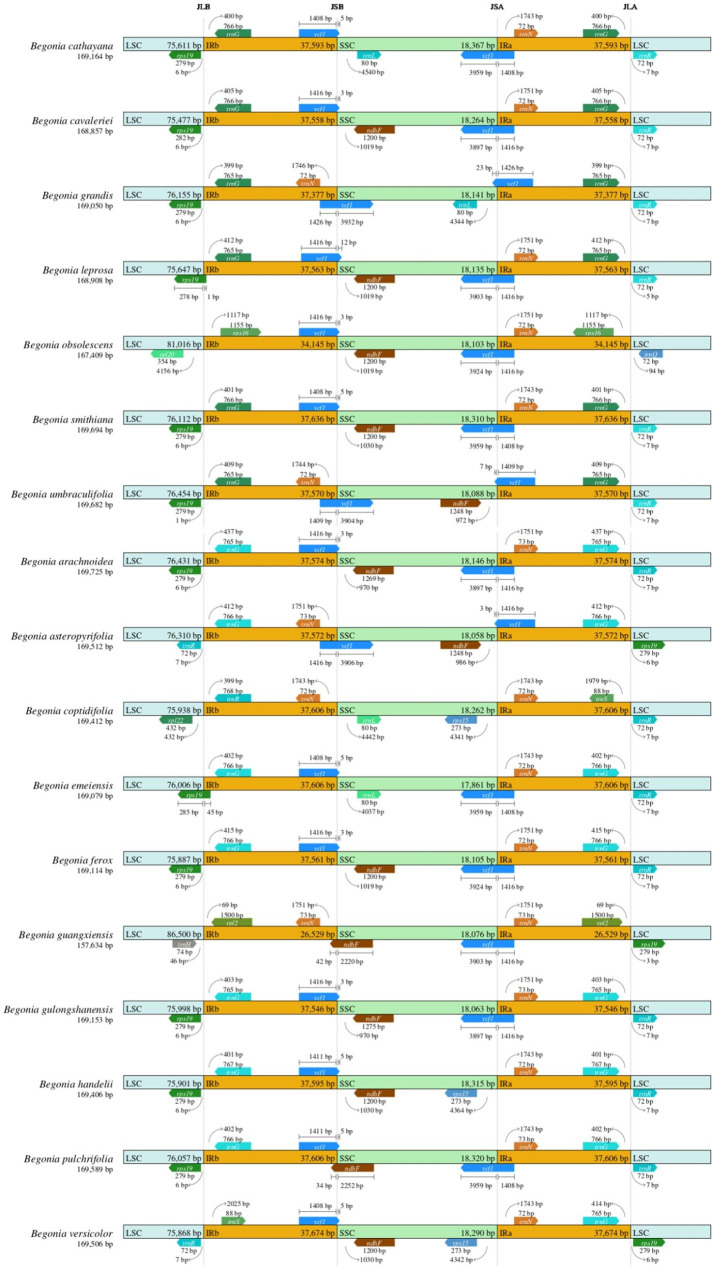
Comparison of the borders of the LSC, SSC, and IR regions among seventeen *Begonia* chloroplast genomes

In addition, the cp. genome had four borders among LSC, IRb, IRa, and SSC: the JLB line for the LSC/IRb border, the JSB line for the IRb/SSC border, the JSA line for the SSC/IRa border, and the JLA line for the IRa/LSC border. The borders of the seventeen cp. genomes of *Begonia* were compared (Fig. 2). At the LSC/IRb boundary, the *rps*19 and *trn*G genes were located at the JLB line in all species except *B. leprosa* and *B. obsolescens*, where the *rps*19 gene was completely located in the LSC region, 1–6 bp from the LSC/IRb boundary, due to the expansion of the LSC region boundary. The IRb/SSC boundary exhibited greater variability. In *B. guangxiensis* and *B. pulchrifolia*, the JSB line was located at the overlap of the *ycf*1 and *ndh*F genes. In *B. obsolescens*, the JSB line was located to the left of the *ycf*1 pseudogene, approximately 4 bp away, whereas in other species, the JSB line was located within the *ycf*1 pseudogene, extending 3–23 bp into the SSC region. The *ycf*1 genes were also observed at the SSC/IRa boundary and contained a segment ranging from 1,408 to 1,426 bp within the IRa region. At the IRa/LSC boundary, all species, except *B. obsolescens* and *B. guangxiensis*, the *trn*G and *trn*R genes located within the boundaries of the JLA line. There were 5–7 bp between *trn*R and the LSC/IRb borders. Additionally, the copy genes of *trn*G were found to be fully retained within the IRa region and positioned at a distance of 399–437 bp from the JLA line.

### Repeat sequence analysis

**Fig. 3:**
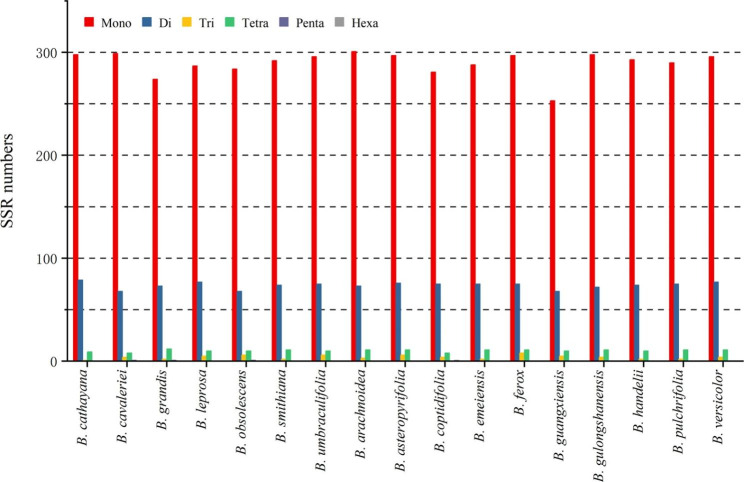
The number of different types of SSRs in seventeen *Begonia* chloroplast genomes

In cp. genomes, repetitive sequences are essential for genome evolution and rearrangements. 897 SSRs were found in the genomes of the seventeen *Begonia* chloroplast genomes (Fig. 3 and Supplementary Table S1). The least number of SSRs (48) was found in the 17 chloroplast genomes of *B. ferox*, *B. gulongshanensis*, and *B. versicolor.* A total of 63 SSRs were discovered in the *B. emeiensis* chloroplast genome, which accounts for the majority of the 17 chloroplast genomes. For each species of *Begonia*, mononucleotide repeats ranged from 39 to 56, while dinucleotide repeats ranged from 5 to 7, tetranucleotide repeats ranged from 0 to 1, hexanucleotide repeats ranged from 0 to 1, trinucleotide repeats ranged from 0 to 1, and pentanucleotide repeats are 0.

Furthermore, oligonucleotide repeat analysis revealed that the seventeen cp. genomes have varying numbers of repeat types and random permutations. Most repeat sequences were within 30–36 bp (Supplementary Table S2). Meanwhile, palindromic repeat (P-repeat) and forward repeat (F-repeat) occurred more frequently than Reverse repeat (R-repeat) and Complement repeat (C-repeat). Supplementary Table S2 displays the structural analysis of the repeat sequence. *B. pulchrifolia* had the fewest repetitions (24), whereas *B. umbraculifolia, B. arachnoidea*, and *B. gulongshanensis* had the most repetitions (36). The examination of repeat sequences will be useful for understanding genetic diversity in *Begonia*.

### Comparative chloroplast genome analysis

**Fig. 4:**
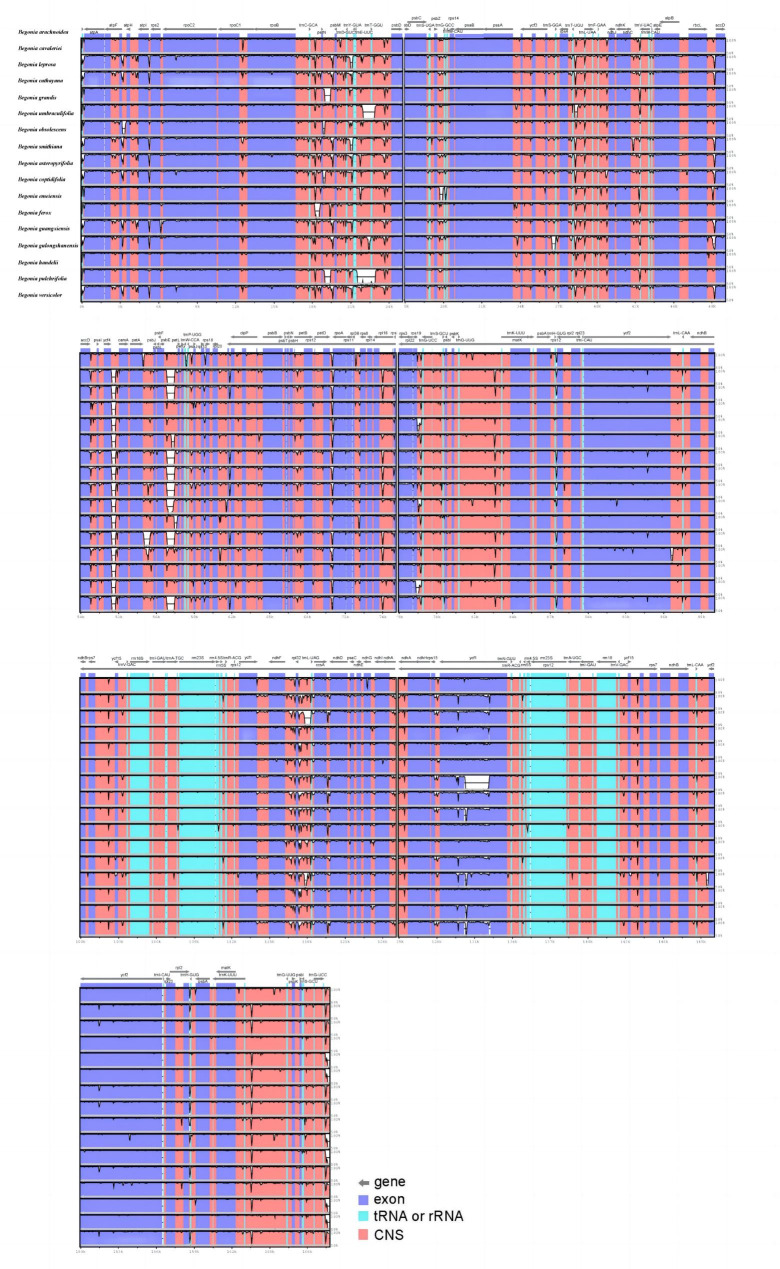
The comparative analysis with LAGAN program of the whole-chloroplast genome of seventeen different species of *Begonia.* The x-axis represents the coordinate in the chloroplast genome

**Fig. 5:**
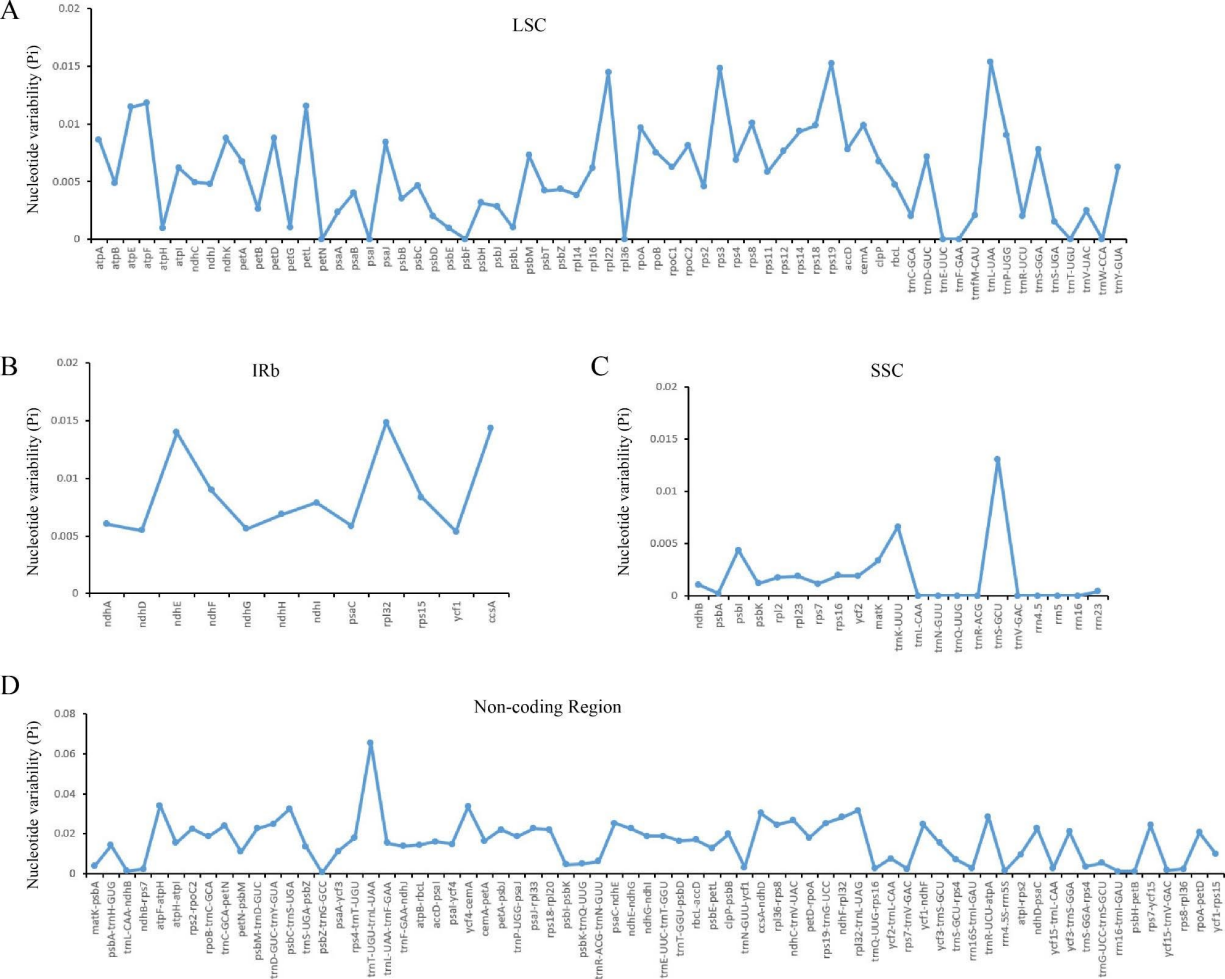
The nucleotide diversity (Pi) value in the seventeen *Begonia* chloroplast genomes. (**A**) The Pi value of LSC region. (**B**) The Pi value of IRb region. (**C**) The Pi value of SSC region. (**D**) The Pi value of Non-coding region

Multiple alignments of the genomes of 17 different species of *Begonia* were performed using *B. coptidifolia* as a reference for comparison. The results revealed that the non-coding regions had more divergence than the coding regions (Fig. 4). In the chloroplast genome alignment, we observed sixteen variant gaps, with 12 regions (*atp*H-*atp*I, *atp*F-*atp*H, *trn*C-GCA-*pe*tN, *pet*N-*psb*M, *ycf*4-*cem*A, *rbc*L-*acc*D, *psb*E*-pet*L, *pet*D-*rpo*A, *rps*19-*trn*G-UCC, *trn*T-UGU-*trn*L-UAA, *trn*E-UUC-*trn*T-GGU, and *trn*D-GUC-*trn*Y-GUA) identified as the primary sources of divergence in the non-coding regions, while *atp*F, *pet*L, *ycf*1, and *ndh*F were identified as the substantially divergent sequences for the coding regions (Fig. 5). Notably, the nucleotide sequences of these regions differed by more than 50% from those of the reference species, *B. coptidifolia*. These sequences may be useful for identifying the species of *Begonia*. We further investigated sequence variability by calculating nucleotide polymorphisms (Pi) among the 17 species of *Begonia*. The results revealed that the coding regions of the SSC region exhibited the highest average Pi values (Fig. 5A), followed by the coding regions of the IR and LSC regions (Fig. 5B C). Among the 12 spacer regions analyzed, the Pi values ranged from 0.01084 (*pet*N*-psb*M*)* to 0.06525 (*trn*T-UGU-*trn*L-UAA) (Fig. 5D). The ten coding genes with the highest polymorphisms were *rps*19, *rps*3, *rpl*32, *rpl*22, *ccs*A, *ndh*E, *atp*F, *pet*L, *atp*E, and *rps*8 (Pi > 0.01). Additionally, tRNA and rRNA gene Pi values were computed. The findings revealed that *trn*L-UAA had a higher Pi value, which was 0.01538. Finally, we identified six highly variable loci with Pi values ranging from 0.03 to 0.07. These loci include *trn*T-UGU-*trn*L-UAA (Pi = 0.06525), *atp*F-*atp*H (Pi = 0.03402), *ycf*4-*cem*A (Pi = 0.03353), *psb*C-*trn*S-UGA (Pi = 0.03245), *rpl*32-*trn*L-UAG (Pi = 0.03162), and *ccs*A-*ndh*D (Pi = 0.03027) (detailed information listed in Supplementary Table S3). These highly variable regions hold the potential to serve as molecular markers for DNA barcoding applications.

### Phylogenetic relationship

**Fig. 6:**
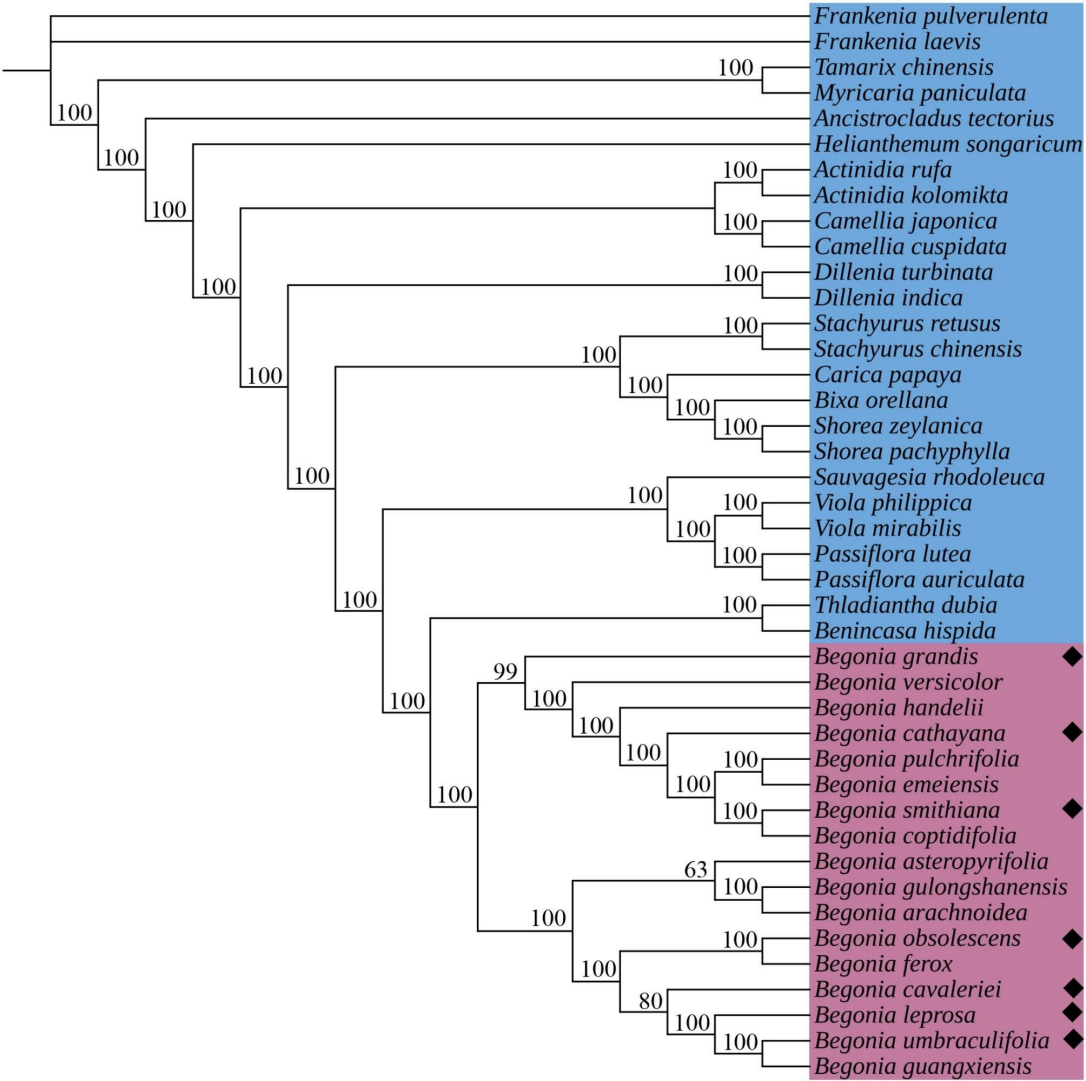
The ML phylogenetic tree of 42 species. Supporting values of > 50% for ML were shown on the branch. 25 species as the outgroup were colored blue, 17 *Begonia* species were colored purple ◆— newly sequenced species

In order to explore the phylogenetic position of this genus, ML trees were built using 76 protein-coding genes from the chloroplast genomes of 42 species, including 17 species of *Begonia* (Fig. 6), among which 35 of 42 species’ chloroplast genomes were obtained from NCBI (Supplementary Table S4). All nodes in the phylogenetic tree had bootstrap values that were more than 50% supported, and each genus formed a clade (bootstrap values of 100%). Two significant minor clades within the *Begonia* clade were separated with 100% bootstrap support. In one major small clade, *B. coptidifolia* and *B. smithiana* form a clade, and then sequentially form clades with *B. emeiensis, B. pulchrifolia, B. cathayana, B. handelii, B. versicolor, and B. grandis.* In another major clade, the clade formed by *B. guangxiensis* and *B. ummbraculifolia* clustered with *B. cavaleriei*, *B. ferox, B. obsolescens* and then shared a sister relationship with *B. asteropyrifolia, B. arachnoidea*, and *B. gulongshanensis.*

### Adaptive evolution analysis

**Fig. 7:**
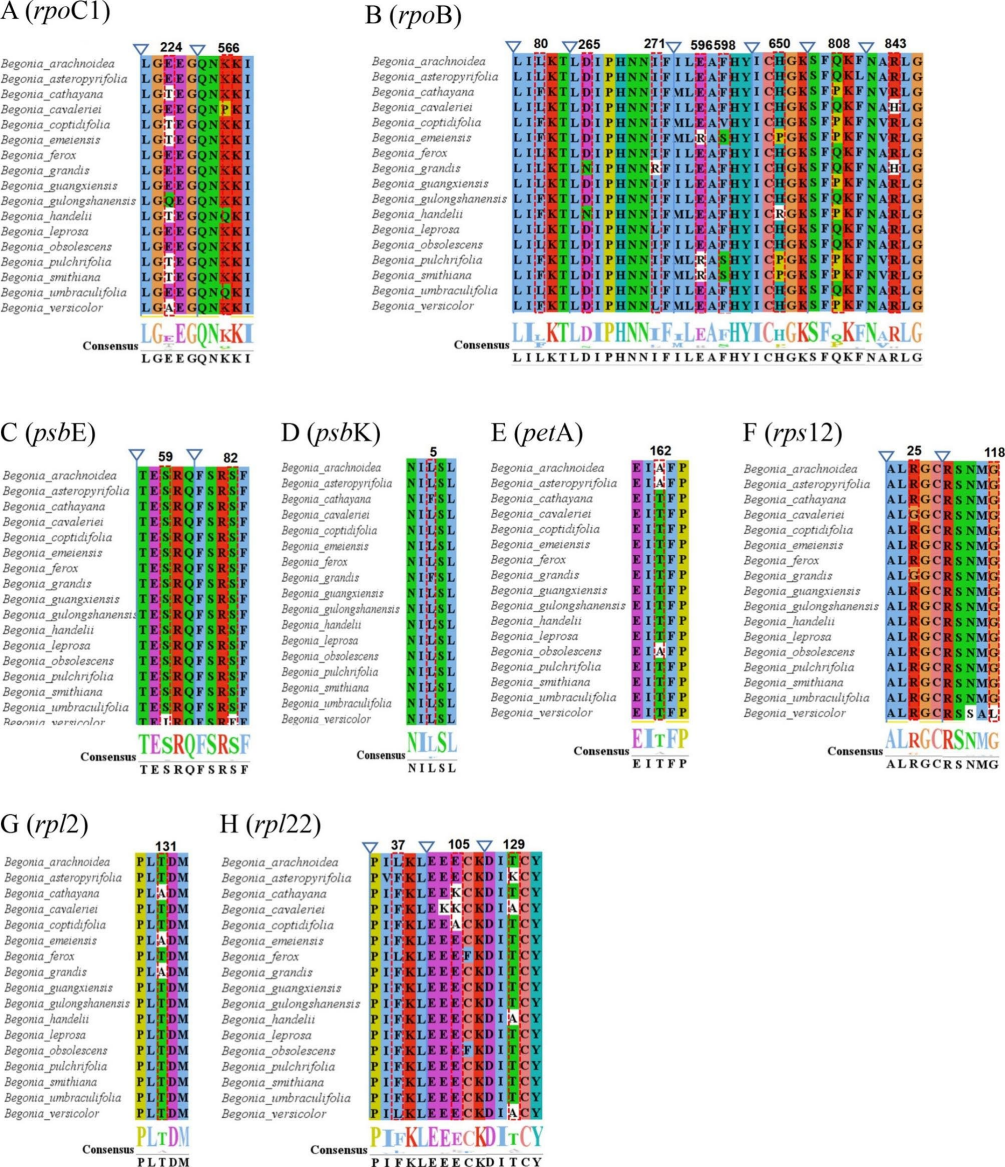
Eight genes of positive selection of amino acid sequences in site model tests

**Fig. 8:**
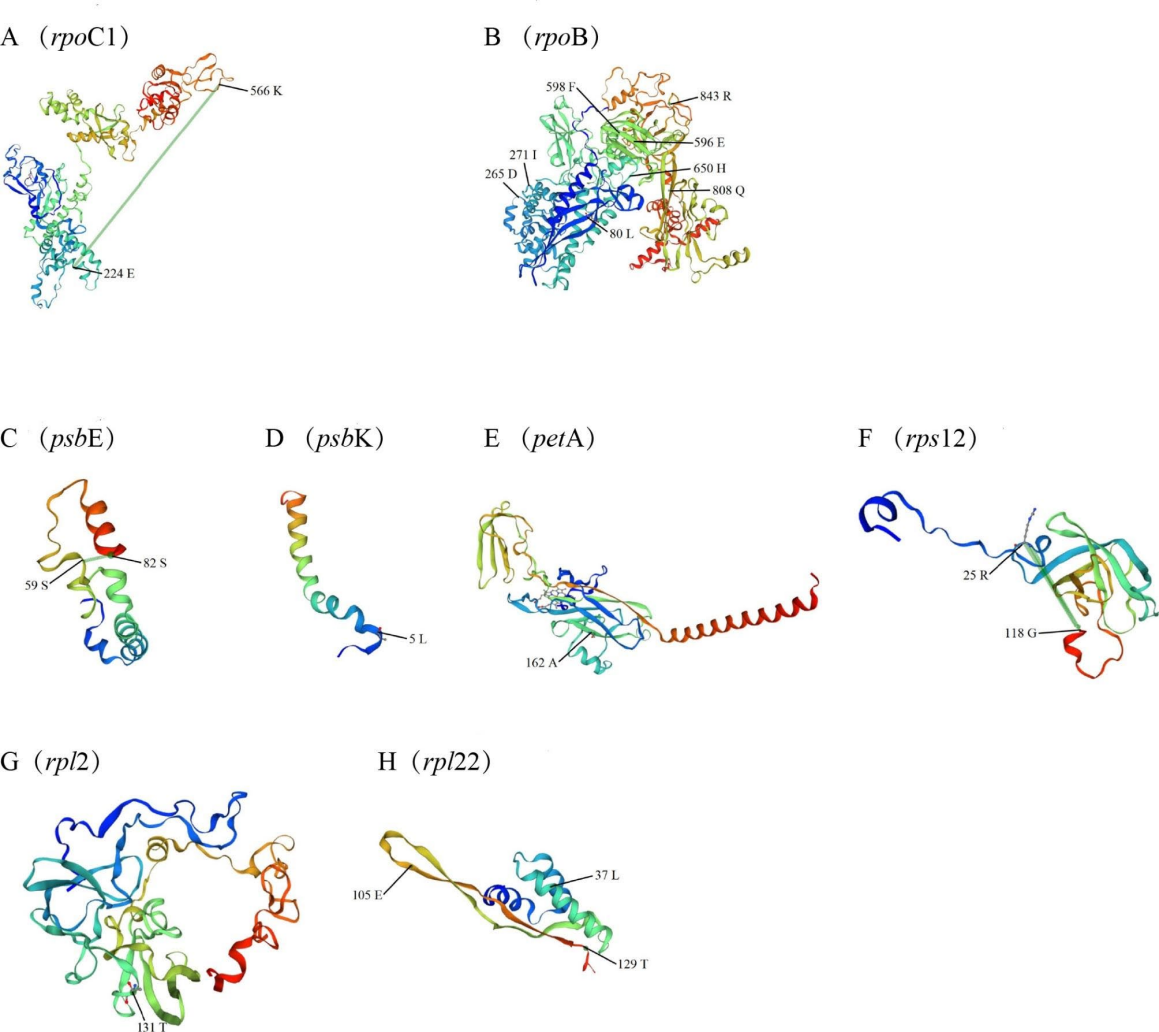
Spatial location of the positively selected sites in proteins of *B. cathayana.* (**A**) Spatial location of the positively selected sites in the *rpo*C1 protein of *B. cathayana.* (**B**) Spatial location of the positively selected sites in the *rpo*B protein of *B. cathayana.* (**C**) Spatial location of the positively selected sites in the *psb*E protein of *B. cathayana.* (**D**) Spatial location of the positively selected sites in the *psb*K protein of *B. cathayana.* (**E**) Spatial location of the positively selected sites in the *pet*A protein of *B. cathayana.* (**F**) Spatial location of the positively selected sites in the *rps*12 protein of *B. cathayana.* (**G**) Spatial location of the positively selected sites in the *rpl*2 protein of *B. cathayana.* (**H**) Spatial location of the positively selected sites in the *rpl*22 protein of *B. cathayana*

Using the BEB test, positive selection was discovered in eight genes (*psb*E, *psb*K, *rpl2*, *rpl22*, *rpoC*1, *pet*A, *rps12*, and *rpo*B) with a high posterior probability (> 95%) in the 76 cp. genome protein-coding genes of seventeen species of *Begonia* (Fig. 7). RNA polymerase subunit coding genes are represented by the *rpo*C1 and *rpo*B genes. Two amino acid positions (224th and 566th codons) in the *rpo*C1 gene were identified as positive selection sites (Fig. 7A). The two sites were discovered through a spatial analysis to be in random coils (Fig. 8A). In *rpo*B, seven sites were found (Fig. 7B). As predicted by the protein structure, the majority of these positive selection sites were found in the α-helix, followed by random coils and the β-sheet (Fig. 8B). The *psb*E and *psb*K genes, which code for the photosystem II subunit, as well as the *pet*A gene, which codes for the cytochrome b/f complex subunit protein in the photosystem II process, make up three of the eight genes. The 59th and 82nd amino acid positions in *psb*E, which were favorably chosen, were situated in the random coil and α-helix, respectively (Fig. 7C and Fig. 8C). The fifth amino acid location in Maturase, which is encoded by the *psb*K gene, was found to be positively selected (Fig. 7D). The locus was discovered through spatial analysis to be inside a random coil (Fig. 8D). The protein structure predicted that *pet*A would have a positively chosen amino acid location (162nd) in the α-helix (Fig. 7E and Fig. 8E).

At the same time, the other three genes were protein synthesis-encoding genes, which were the ribosomal protein small subunit (RPS) *rps*12 gene; the *rpl*2 and *rpl*22 genes of the ribosomal protein large subunit (RPL). In the *rps*12 protein, two regions of positive selection (25th and 118th) were discovered. The *rps*12 protein under positive selection’s spatial analysis revealed that the location was in the α-helix (Fig. 7F and Fig. 8F). Figures 7G and 8G show that the *rpl*2 protein has a positive selection site (131st), Fig. 7H and Fig. 8H show that the random coil also contains three amino acid sites under positive selection in *rpl*22 (37th, 105th, and 129th).

## Discussion

### Plastid genome features

This study described the genomes of seventeen distinct species of *Begonia* (Fig. 1; Table 1). In terms of structures, gene order, gene numbers (protein-coding genes, rRNAs, and tRNAs), and intron number, the seventeen cp. genomes displayed a highly conserved feature. The length of the cp. genomes in seventeen different *Begonia* plants ranged from 157,634 to 169,725 bp. Although it is thought that the major reason for the cp. genome’s size fluctuation is the contraction and extension of the border between the IR and SSC regions [6], the cp. genomes of angiosperms have a highly conserved feature. The seventeen chloroplast genomes of *Begonia* showed similar behavior. Despite the 17 chloroplast genomes of *Begonia* having well-conserved genomic structure, including gene number and order, length variations of the whole sequences that make up the IR, LSC, and SSC regions were discovered among these chloroplast genomes (Fig. 2). The IRb/SSC boundary exhibited greater variability, showed the most variation in location and length across the seventeen chloroplast genomes of Begonia species. The *ycf*1, *trn*G and *trn*R sequences alteration may be brought on by the border contraction and expansion between the IRb/SSC regions in plants [9].

### Repeat sequence analysis

SSRs have been used as molecular markers to find significant levels of variation in species that are closely related to one another and to help analyze population genetics and polymorphisms [10]. There were 897 SSRs in the seventeen chloroplast genomes, with 777 of them being mono-nucleotide repeats, which make up the majority of SSRs (86.62%) (Fig. 3). Among the seventeen chloroplast genomes, the most mono-nucleotide repeats were discovered. Many more studies with comparable findings in angiosperm cp. genomes have also been published [11, 12]. In accordance with the overall SSR features of chloroplast genomes in angiosperms, the findings also revealed that polyadenine (Poly-A) or polythymine (Poly-T) repeats predominated the SSRs detected in the cp. genome, with low quantities of polyguanine (Poly-G) and polycytosine (Poly-C) repeats [13, 14].

In addition, 1,261 non-overlapping sequence repetitions were found in seventeen chloroplast genomes (Fig. 4), with *B. obsolescens* having the highest number (88) and *B. emeiensis* having the lowest number (53). There were 1,261 repetitions in all, divided into four categories: palindromic repetition, complement repetition, forward repetition, and reverse repetition. Palindromic and forward repetition accounted for the greatest proportion. Analysis of repeated regions in the chloroplast genomes of other plants led to the same result [15]. These SSRs and non-overlapping sequence repeats may all be used to create markers for the genetic diversity analyses of different species of *Begonia* (Supplementary Table S1).

### Phylogenetic relationship

The majority of research on the molecular phylogeny of *Begonia* was based on chloroplast (*psb*A*-trn*H, *mat*K, and *rbc*L) sequences and nuclear (ITS and ITS2) fragments. However, the phylogenetic relationship of *Begonia* was not completely resolved by these sequences [16, 17]. Plant phylogeny based on the whole chloroplast genome has become popular in recent years [18, 19]. In this work, 42 species from 18 genera and 82 protein-coding genes from the chloroplast genomes were used to create the ML tree (Fig. 6). A clade consisting of all 17 species of *Begonia* was detected using 100% bootstrap values. The phylogenetic relationship of *Begonia* could be completely explained by the sequences of 82 protein-coding genes, and the topology of the phylogenetic tree generated in this study was compatible with the findings of earlier investigations [1, 4]. More genetic information on the phylogeny and evolutionary relationships between these species may be available from chloroplast genome data.

### Adaptive evolution analysis

The plants of *Begonia* are mainly distributed in shady areas of south and southwest China. Light has a regulatory role in plant growth and development, especially in the differentiation of young plants. In addition, light promotes the growth of the roots, stems, and leaves of the plant and can effectively prevent it from growing. In most cases, insufficient light weakens photosynthesis, causing the plant to grow or the leaves to turn yellow, inhibiting root growth. The plant is poorly exposed to light, flower buds are not easily formed or stunted, and fruit development is hindered, resulting in flower and fruit drops. Most *Begonia* species are shade-tolerant plants, but their adaptation to shade varies: deep shade plants such as *B. umbraculifolia* and *B. guangxiensis* can be found in tropical rainforests and the interiors of karst limestone caves, respectively. *B. coptidifolia* and *B. smithiana*, on the other hand, are adapted to semi-shaded and more open habitats. This shows that species of *Begonia* adapted to similar environments are also consistent with phylogenetic relationships (Fig. 6). These challenging shady environments may impose selective pressure on genes, which might leave a natural selection mark on chloroplast genes involved in environmental adaptation. In this work, eight genes (*rpo*C1, *rpo*B, *psb*E, *psb*K, *pet*A, *rps*12, *rpl*2, and *rpl*22) were found to be under positive selection using a site model among the chloroplast genes of seventeen species of *Begonia* (Fig. 7and Fig. 8).

The *psb*E, *psb*K, and *rbc*L genes were involved in photosynthesis and were being positively selected. Photosystem II subunits E and K are encoded by the *psb*E and *psb*K genes, respectively. The initial stage of photosynthesis begins with Photosystem II, which uses photons to remove electrons from water molecules [20]. While not required for the construction or operation of the photosystem II complex, *psb*E and *psb*K are crucial for the best possible performance of photosystem II. The *psb*K gene was found to be under positive selection in *Echinacanthus*, *Calligonum*, and *Mongolicum*, among other plants, and is thought to be a key player in the process of plants adapting to their varied environments [21].

Additionally, positive selection sites in the *rpl*2, *rpl*22, and *rps1*2 genes were discovered. The large ribosomal subunit proteins are encoded by the *rpl* genes [22]. The translation of chloroplast ribosomes may be impacted by the mutation of genes encoded in ribosomal proteins under the pressure of the natural environment [23].

In the *rpo*C1 and *rpo*B genes, one site and eight sites were found to be under positive selection, respectively. The adaptation of the *Allium genus* (Amaryllidaceae) was also discovered to involve positive selection of *rpo*C1 and *rpo*B [24]. Changes to the *rpo*B gene in *Mycobacterium tuberculosum* mutations may affect the ability of the bacteria to survive in environments with high oxygen levels [25]. Under aerobic growth conditions, the guanine base is transformed into 8-oxo-7,8-dihydroguanine in *Escherichia coli* cells. Both adenine and cytosine can combine with this substance. As a result, the effectiveness of RNA polymerases determines how accurately RNA is synthesized, and in aerobic settings, substitutions are made in the subunits, which are encoded by the *rpo*C and *rpo*B genes [26]. These facts support the idea that highly oxygenic environments, such as caves and habitats with deep shade, can exert selective pressure on the function of RNA polymerase in chloroplasts, changing the protein sequence to produce stable function in environments with a lot of active oxygen produced by overloaded Photosystem II.

Photosynthesis-related genes are more likely to have evolved adaptively in plants living in shady conditions, such as aquatic or shade plants [27]. Environmental characteristics, including light intensity, soil water content, and nutrient availability, fluctuate greatly in karst areas, which may have had a significant selection impact on plant development [28]. In this study, eight chloroplast genes (*rpo*C1, *rpo*B, *psb*E, *psb*K, *pet*A, *rps*12, *rpl*2, and *rpl*22) that were being positively selected, the majority of which were involved in photosynthesis and protein synthesis, may have helped *Begonia* species evolve and adapt to shady extreme conditions.

## Conclusion

This study presented and compared the cp. genomes of 17 different *Begonia* species, including the recently sequenced cp. genomes of seven *Begonia* species. The genome annotation and comparison analysis revealed that each chloroplast genome had a typical quadripartite structure, comparable to that of classic angiosperms, and that their GC contents, gene numbers, and gene orders were identical. The structure, makeup, and gene order of the seventeen species of *Begonia* were all comparable in their cp. genomes. There were a total of 1,261 non-overlapping sequence repeats and 897 SSRs found in the seventeen chloroplast genomes of *Begonia* species, which were valuable sources for creating markers for genetic diversity research. Furthermore, we found that six different regions (*trn*T-UGU-*trn*L-UAA, *atp*F-*atp*H, *ycf*4-*cem*A, *psb*C-*trn*S-UGA, *rpl*32-*trn*L-UAG, *ccs*A-*ndh*D) were potential molecular markers in seventeen *Begonia* species. The genetic and evolutionary relationships of 40 species belonging to 16 genera were clearly shown by the phylogenetic tree constructed using 82 protein-coding genes. Eight chloroplast genes showed signs of positive selection, according to adaptive evolution analyses (i.e., *rpo*C1, *rpo*B, *psb*E, *psb*K, *pet*A, *rps*12, *rpl*2, and *rpl*22). Changes in these positive selection sites may have influenced *Begonia*’s evolution to withstand harsh habitats in shady conditions. The establishment and use of germplasm resources for *Begonia* species as well as the creation of conservation strategies will be made possible by these investigations of chloroplast genomes.

## Materials and methods

### Ethics approval and consent to participate

For the collection of samples for this study, no special licenses were needed. The relevant Chinese laws were followed as this research was conducted.

### Plant materials and DNA extraction

The seven species of *Begonia* were collected from the Guangxi Institute of Botany, and all these samples were identified by Shizhong Mao, who is an associate researcher at the Guangxi Institute of Botany, Chinese Academy of Sciences. All voucher specimens were deposited in the School of Life Science and Technology, Wuhan Polytechnic University, and detailed sample information is listed in Table 3. The total genomic DNA was extracted from fresh leaves using the TianGen DNA extraction kit according to the manufacturer’s protocol. To make sure the genomic DNAs satisfied the criteria for sequencing, their quality and concentration were assessed using the Qubit2.0 Fluorometer (Thermo Scientific, USA) and NanoDrop 2000c spectrophotometer (Nanodrop Technologies, Wilmington, DE, USA).

**Table 3:** Sources of material from seven *Begonia* species

Taxon	Voucher	GenBank number	Location	Habitat
*B. cathayana*	GX202201	OP618124	Fangchenggang Guangxi, China	Forests, slopes and valleys, gully bottoms in deep shady; ca. 1,200-1,500 m
*B. cavaleriei*	GX202202	OP618122	Baise Guangxi, China	Ravines, foot of the mountains, valleys in deep shady; ca. 700-1,000 m
*B. grandis*	GX202203	OP618125	Nanning Guangxi, China	Wet stone, densely forested rocks in valleys, ca. 100-1,100 m
*B. leprosa*	GX202204	OP618123	Hechi Guangxi, China	Undergrowth, roadsides and hillsides in semi-shady, ca. 700-1,800 m
*B. obsolescens*	GX202205	OP618127	Liuzhou Guangxi, China	Rocky crevices; ca. 1,200 m
*B. smithiana*	GX202206	OP618128	Hechi Guangxi, China	Gullies, valleys, foot of the mountains; ca. 700-1,320 m
*B. umbraculifolia*	GX202207	OP618126	Nanning Guangxi, China	Limestone rocks, understory or valley; ca. 170–500 m

### Library construction, genome sequencing and assembling

Firstly, according to the library instructions, qualified and purified genomic DNA was used to construct the sequencing library. Then the total DNA was sequenced using the Illumina HiSeq 2000 sequencer (Illumina Biotechnology Company, San Diego, CA, USA) [29], and the paired-end (PE) was read at 350 bp. Secondly, raw data were trimmed and filtered by the NGS QC Toolkit (v2.3.3) [30] to get clean data. Thirdly, the cp. genome was assembled from clean data using NOVOPlasty (v2.7.2) with a k-mer length of 39 bp using *B. arachnoidea* (NC_063512) as the reference chloroplast genome sequence. Fourthly, the Bowtie2 (v2.0.1) software was used to map the raw reads to the assembled sequences to verify the correctness of the assembly [31]. Finally, the complete chloroplasts of seven *Begonia* species were obtained (accession numbers: OP618122-OP618128).

### Genome annotation and sequence characterization

CPGAVAS2 [32] was used for seven *Begonia* chloroplast genome annotations based on the *B. arachnoidea* (NC_063512) chloroplast genome by manual adjustment of the positions of start and stop codons. tRNA scan-SE (v1.21) [33] was used to confirm tRNA genes. OrganellarGenomeDRAW (v.1.3.1) was used to create a circular map of seven *Begonia* plastomes [34].

### Repeat sequence analysis

SSRs in the cp. genomes were identified by the Perl script MISA (http://pgrc.ipk-gatersleben.de/misa/) with specific parameters: ≥ 10 for mono-, ≥ 8 for di-, ≥ 4 for tri- and tetra-, ≥ 3 for penta- and hexa- [35]. The forward (F), reverse (R), complement (C), and palindromic (P) long repeat sequences in the cp. genomes were analyzed using REPuter with the following parameters: a minimum repeat size of 30 bp and a Hamming distance of 3 [36].

### Genome comparative analysis

In order to identify genome structure, gene content, genome size, and repeat variations, all seventeen *Begonia* species were compared. Firstly, the whole cp. genome sequences of the seventeen *Begonia* species were aligned using the shuffle-LAGAN mode in mVISTA, in which the cp. genome annotated in *B. arachnoidea* (NC_063512) was used as a reference [37]. Secondly, the borders of LCS, SSC, and IR in the cp. genomes of seventeen *Begonia* species were compared using Irscope software [38]. Thirdly, the percentage of variable sites in the protein-coding genes was analyzed using MEGA 6.0 [39]. Finally, DnaSP (v. 6.0) was used to calculate Pi among seventeen *Begonia* chloroplast genomes [40].

### Phylogenetic analysis

For phylogenetic analysis, 42 species (17 *Begonia* species and 25 other species) were used, with 35 chloroplast genomes obtained from NCBI (detailed information list in Supplementary Table S4). These sequences were aligned with MAFFT (v 7.222) [41] and then used to construct the phylogenetic trees using the maximum likelihood (ML) method in RAxML (v 7.0.4) [42]. To assess branch support, the ML tree was built using the GTR + CAT model and 1000 bootstrap replicates.

### Adaptive evolution analysis

To assess the genes in the cp. genomes of *Begonia* that underwent positive selection, DnaSP (v6.0) was used to calculate the non-synonymous (dN), synonymous (dS), and dN/dS (ω) values of protein-coding genes. The CDS sequences were extracted from the chloroplast genomes of *Begonia*, and then the single-copy CDS sequences common to all *Begonia* species were selected and aligned under the codon model. The positive selection sites were identified by using EasyCodeML (v1.21) [20] software with the following parameters: seqtype = 1, model = 0, NSsites = 0, 1, 2, 3, 7, 8. Then, the posterior probabilities (> 0.95) for amino acid positions that might be subject to positive selection were determined using the BEB method [43]. Additionally, the LRT was used to evaluate the logarithmic likelihood value of site models and their statistical significance. Finally, the amino acid sequences of the positively chosen genes’ secondary structure were visualized using the PSIPRED website [44], and the proteins for these genes were predicted using the online SWISS-MODEL program [45].

### Electronic supplementary material

Below is the link to the electronic supplementary material.

**Supplementary Table S1** Summary of SSRs in seventeen *Begonia* chloroplast genomes.

## Data Availability

The datasets generated and analyzed in this study are available in the GenBank of NCBI, and the complete chloroplast genome sequence of seven *Begonia* species is deposited in GenBank of NCBI under accession number OP618122-OP618128. The accession numbers for the remaining datasets used and analyzed in this study are listed in the Methods section and Supplementary Tables section.

## Abbreviations

cp: Chloroplast
LSC: Large single copy
SSC: Small single copy
IR: Inverted repeat
PolyA: Polyadenine
PolyT: Polythymine
ML: Maximum likelihood
Pi: Nucleotide diversity
Ka: Non-synonymous substitutions per non-synonymous site
Ks: Synonymous substitutions per synonymous site
CDS: Cp-coding sequences
rRNA: Ribosomal RNA
tRNA: Transfer RNA
SSR: Simple sequence repeat
