# Supplementary material for: Comparative chloroplast genomics reveals the phylogeny and the adaptive evolution of Begonia in China
Source: BMC Genomics. 2023 Oct 27;24:648. doi: 10.1186/s12864-023-09563-3 (PMC10612195; doi:10.1186/s12864-023-09563-3)
Supplement: Supplementary file 1 — Supplementary Table S1 Summary of SSRs in seventeen Begonia chloroplast genomes. [file 12864_2023_9563_MOESM1_ESM.doc]

**Supplementary Table S1** Summary of SSRs in seventeen *Begonia* chloroplast genomes.

| **Species** | **Mono** | **Di** | **Tri** | **Tetra** | **Penta** | **Hexa** | **Total** |
| --- | --- | --- | --- | --- | --- | --- | --- |
| *B. cathayana* | 47 | 6 | 1 | 1 | 0 | 0 | 55 |
| *B. cavaleriei* | 43 | 6 | 1 | 1 | 0 | 0 | 51 |
| *B. grandis* | 39 | 7 | 1 | 1 | 0 | 1 | 49 |
| *B. leprosa* | 45 | 5 | 1 | 1 | 0 | 0 | 52 |
| *B. obsolescens* | 46 | 5 | 1 | 1 | 0 | 0 | 53 |
| *B. smithiana* | 54 | 6 | 1 | 0 | 0 | 0 | 61 |
| *B. umbraculifolia* | 50 | 6 | 0 | 0 | 0 | 0 | 56 |
| [*B. arachnoidea*](https://www.ncbi.nlm.nih.gov/nuccore/NC_063512.1) | 41 | 6 | 1 | 0 | 0 | 1 | 49 |
| [*B. asteropyrifolia*](https://www.ncbi.nlm.nih.gov/nuccore/NC_065014.1) | 48 | 6 | 1 | 0 | 0 | 0 | 55 |
| [*B. coptidifolia*](https://www.ncbi.nlm.nih.gov/nuccore/NC_056110.1) | 45 | 5 | 0 | 1 | 0 | 0 | 51 |
| [*B. emeiensis*](https://www.ncbi.nlm.nih.gov/nuccore/NC_061410.1) | 56 | 6 | 1 | 0 | 0 | 0 | 63 |
| *B. ferox* | 42 | 5 | 0 | 1 | 0 | 0 | 48 |
| [*B. guangxiensis*](https://www.ncbi.nlm.nih.gov/nuccore/NC_046385.1) | 46 | 5 | 0 | 1 | 0 | 0 | 52 |
| [*B. gulongshanensis*](https://www.ncbi.nlm.nih.gov/nuccore/NC_063513.1) | 45 | 6 | 1 | 0 | 0 | 1 | 53 |
| [*B. handelii*](https://www.ncbi.nlm.nih.gov/nuccore/NC_065245.1) | 41 | 6 | 1 | 0 | 0 | 0 | 48 |
| *B. pulchrifolia* | 47 | 5 | 0 | 1 | 0 | 0 | 53 |
| *B. versicolor* | 42 | 5 | 0 | 1 | 0 | 0 | 48 |
| Total | 777 | 96 | 11 | 10 | 0 | 3 | 897 |

**Supplementary Table S2** Summary of complex repeats in seventeen *Begonia* chloroplast genomes.

| **Species** | **Palindromic**  **(P)** | **Forward**  **(F)** | **Reverse**  **(R)** | **Complement**  **(C)** | **Total** |
| --- | --- | --- | --- | --- | --- |
| *B. cathayana* | 28 | 28 | 9 | 8 | 73 |
| *B. cavaleriei* | 28 | 28 | 9 | 6 | 71 |
| *B. grandis* | 33 | 28 | 2 | 2 | 65 |
| *B. leprosa* | 35 | 34 | 7 | 4 | 80 |
| *B. obsolescens* | 32 | 46 | 7 | 3 | 88 |
| *B. smithiana* | 34 | 37 | 6 | 4 | 81 |
| *B. umbraculifolia* | 36 | 31 | 5 | 5 | 77 |
| [*B. arachnoidea*](https://www.ncbi.nlm.nih.gov/nuccore/NC_063512.1) | 36 | 39 | 5 | 4 | 84 |
| [*B. asteropyrifolia*](https://www.ncbi.nlm.nih.gov/nuccore/NC_065014.1) | 31 | 29 | 3 | 2 | 65 |
| [*B. coptidifolia*](https://www.ncbi.nlm.nih.gov/nuccore/NC_056110.1) | 36 | 35 | 7 | 4 | 82 |
| [*B. emeiensis*](https://www.ncbi.nlm.nih.gov/nuccore/NC_061410.1) | 24 | 23 | 4 | 2 | 53 |
| *B. ferox* | 34 | 36 | 8 | 4 | 82 |
| [*B. guangxiensis*](https://www.ncbi.nlm.nih.gov/nuccore/NC_046385.1) | 31 | 30 | 3 | 3 | 67 |
| [*B. gulongshanensis*](https://www.ncbi.nlm.nih.gov/nuccore/NC_063513.1) | 36 | 36 | 13 | 2 | 87 |
| [*B. handelii*](https://www.ncbi.nlm.nih.gov/nuccore/NC_065245.1) | 30 | 31 | 8 | 12 | 81 |
| *B. pulchrifolia* | 24 | 23 | 4 | 2 | 53 |
| *B. versicolor* | 35 | 29 | 5 | 3 | 72 |
| Total | 543 | 543 | 105 | 70 | 1,261 |

**Supplementary Table S3** The nucleotide variability (Pi) value in the seventeen *Begonia* chloroplast genomes.

| **Coding Region** | | **Pi Value** | **Non-coding Region** | **Pi Value** |
| --- | --- | --- | --- | --- |
| **LCS** | *atp*A | 0.00863 | *mat*K-*psb*A | 0.00376 |
|  | *atp*B | 0.00485 | *psb*A-*trn*H-GUG | 0.01415 |
|  | *atp*E | 0.01145 | *trn*L-CAA-*ndh*B | 0.00135 |
|  | *atp*F | 0.01182 | *ndh*B-*rps*7 | 0.00223 |
|  | *atp*H | 0.00096 | *atp*F-*atp*H | 0.03402 |
|  | *atp*I | 0.00621 | *atp*H-*atp*I | 0.01527 |
|  | *ndh*C | 0.00494 | *rps*2-*rpo*C2 | 0.02245 |
|  | *ndh*J | 0.00481 | *rpo*B-*trn*C-GCA | 0.01859 |
|  | *ndh*K | 0.00875 | *trn*C-GCA-*pet*N | 0.02412 |
|  | *pet*A | 0.00674 | *pet*N-*psb*M | 0.01084 |
|  | *pet*B | 0.00265 | *psb*M-*trn*D-GUC | 0.02266 |
|  | *pet*D | 0.00875 | *trn*D-GUC-*trn*Y-GUA | 0.02477 |
|  | *pet*G | 0.00103 | *psb*C-*trn*S-UGA | 0.03245 |
|  | *pet*L | 0.01155 | *trn*S-UGA-*psb*Z | 0.01354 |
|  | *pet*N | 0 | *psb*Z-*trn*G-GCC | 0.00043 |
|  | *psa*A | 0.00235 | *psa*A-*ycf*3 | 0.01112 |
|  | *psa*B | 0.00405 | *rps*4-*trn*T-UGU | 0.018 |
|  | *psa*I | 0 | *trn*T-UGU-*trn*L-UAA | 0.06525 |
|  | *psa*J | 0.00839 | *trn*L-UAA-*trn*F-GAA | 0.01545 |
|  | *psb*B | 0.00353 | *trn*F-GAA-*ndh*J | 0.01383 |
|  | *psb*C | 0.00468 | *atp*B-*rbc*L | 0.01441 |
|  | *psb*D | 0.00199 | *acc*D-*psa*I | 0.01607 |
|  | *psb*E | 0.00093 | *psa*I-*ycf*4 | 0.01476 |
|  | *psb*F | 0 | *ycf*4-*cem*A | 0.03353 |
|  | *psb*H | 0.00318 | *cem*A-*pet*A | 0.01646 |
|  | *psb*J | 0.00287 | *pet*A-*psb*J | 0.02195 |
|  | *psb*L | 0.00101 | *trn*P-UGG-*psa*J | 0.01868 |
|  | *psb*M | 0.00728 | *psa*J-*rpl*33 | 0.02265 |
|  | *psb*T | 0.00422 | *rps*18-*rpl*20 | 0.02194 |
|  | psbZ | 0.00436 | *psb*I-*psb*K | 0.00444 |
|  | *rpl*14 | 0.00383 | *psb*K-*trn*Q-UUG | 0.0048 |
|  | *rpl*16 | 0.00621 | *trn*R-ACG-*trn*N-GUU | 0.0062 |
|  | *rpl*22 | 0.01447 | *psa*C-*ndh*E | 0.0253 |
|  | *rpl*36 | 0 | *ndh*E-*ndh*G | 0.0226 |
|  | *rpo*A | 0.0097 | *ndh*G-*ndh*I | 0.01888 |
|  | *rpo*B | 0.00751 | *trn*E-UUC-*trn*T-GGU | 0.01876 |
|  | *rpo*C1 | 0.00624 | *trn*T-GGU-*psb*D | 0.01647 |
|  | *rpo*C2 | 0.00817 | *rbc*L-*acc*D | 0.01689 |
|  | *rps*2 | 0.00455 | *psb*E-*pet*L | 0.01282 |
|  | *rps*3 | 0.01486 | *clp*P-*psb*B | 0.01973 |
|  | *rps*4 | 0.00689 | *trn*N-GUU-*ycf*1 | 0.00315 |
|  | *rps*8 | 0.01006 | *ccs*A-*ndh*D | 0.03027 |
|  | *rps*11 | 0.00587 | *rpl*36-*rps*8 | 0.02447 |
|  | *rps*12 | 0.00766 | *ndh*C-*trn*V-UAC | 0.02658 |
|  | *rps*14 | 0.00937 | *pet*D-*rpo*A | 0.018 |
|  | *rps*18 | 0.00985 | *rps*19-*trn*G-UCC | 0.02518 |
|  | *rps*19 | 0.01528 | *ndh*F-*rpl*32 | 0.02828 |
|  | *acc*D | 0.00782 | *rpl*32-*trn*L-UAG | 0.03162 |
|  | *cem*A | 0.00989 | *trn*Q-UUG-*rps*16 | 0.00244 |
|  | *clp*P | 0.00677 | *ycf*2-*trn*L-CAA | 0.00744 |
|  | *rbc*L | 0.00473 | *rps*7-*trn*V-GAC | 0.00222 |
|  | *trn*C-GCA | 0.00201 | *ycf*1-*ndh*F | 0.0247 |
|  | *trn*D-GUC | 0.00713 | *ycf*3-*trn*S-GCU | 0.01541 |
|  | *trn*E-UUC | 0 | *trn*S-GCU-*rps*4 | 0.00703 |
|  | *trn*F-GAA | 0 | *rrn*16S-*trn*I-GAU | 0.00266 |
|  | *trn*M-CAU | 0.00208 | *trn*R-UCU-*atp*A | 0.02848 |
|  | *trn*L-UAA | 0.01538 | *rrn*4.5S-*rrn*5S | 0.00121 |
|  | *trn*P-UGG | 0.00906 | *atp*I-*rps*2 | 0.00962 |
|  | *trn*R-UCU | 0.00198 | *ndh*D-*psa*C | 0.02257 |
|  | *trn*S-GGA | 0.0078 | ycf15-trnL-CAA | 0.00277 |
|  | *trn*S-UGA | 0.00154 | *ycf*3-*trn*S-GGA | 0.02109 |
|  | *trn*T-UGU | 0 | *trn*S-GGA-*rps*4 | 0.00343 |
|  | *trn*V-UAC | 0.00246 | *trn*G-UCC-*trn*S-GCU | 0.00527 |
|  | *trn*W-CCA | 0 | *rrn*16-*trn*I-GAU | 0.00113 |
|  | *trn*Y-GUA | 0.00628 | *psb*H-*pet*B | 0.00113 |
| **IRb** | *ndh*A | 0.00605 | *rps*7-*ycf*15 | 0.0243 |
|  | *ndh*D | 0.0055 | *ycf*15-*trn*V-GAC | 0.00159 |
|  | *ndh*E | 0.01399 | *rps*8-*rpl*36 | 0.0023 |
|  | *ndh*F | 0.00898 | *rpo*A-*pet*D | 0.02068 |
|  | *ndh*G | 0.00561 | *ycf*1-*rps*15 | 0.00971 |
|  | *ndh*H | 0.00686 |  |  |
|  | *ndh*I | 0.0079 |  |  |
|  | *psa*C | 0.00586 |  |  |
|  | *rpl*32 | 0.01484 |  |  |
|  | *rps*15 | 0.00838 |  |  |
|  | *ycf*1 | 0.00538 |  |  |
|  | *ccs*A | 0.01433 |  |  |
| **SSC** | *ndh*B | 0.00106 |  |  |
|  | *psb*A | 0.00022 |  |  |
|  | *psb*I | 0.00437 |  |  |
|  | *psb*K | 0.00119 |  |  |
|  | *rpl*2 | 0.00176 |  |  |
|  | *rpl*23 | 0.00188 |  |  |
|  | *rps*7 | 0.00113 |  |  |
|  | *rps*16 | 0.00197 |  |  |
|  | *ycf*2 | 0.0019 |  |  |
|  | *mat*K | 0.00333 |  |  |
|  | *trn*K-UUU | 0.00661 |  |  |
|  | *trn*L-CAA | 0 |  |  |
|  | *trn*N-GUU | 0 |  |  |
|  | *trn*Q-UUG | 0 |  |  |
|  | *trn*R-ACG | 0 |  |  |
|  | *trn*S-GCU | 0.01307 |  |  |
|  | *trn*V-GAC | 0 |  |  |
|  | *rrn*4.5 | 0 |  |  |
|  | *rrn*5 | 0 |  |  |
|  | *rrn*16 | 0 |  |  |
|  | *rrn*23 | 0.00043 |  |  |

**Supplementary Table S4** Chloroplast genome sequences from GenBank used in this study.

| **No.** | **Family** | **Species** | **GenBank accession number** |
| --- | --- | --- | --- |
| 1 | Actinidiaceae | *Actinidia kolomikta* | NC_034915 |
| 2 | Actinidiaceae | *Actinidia rufa* | NC_039973 |
| 3 | Ancistrocladaceae | *Ancistrocladus tectorius* | NC_041258 |
| 4 | Begoniaceae | *Begonia arachnoidea* | NC_063512 |
| 5 | Begoniaceae | *Begonia asteropyrifolia* | NC_065014 |
| 6 | Begoniaceae | *Begonia coptidifolia* | NC_056110 |
| 7 | Begoniaceae | *Begonia emeiensis* | NC_061410 |
| 8 | Begoniaceae | *Begonia ferox* | NC_067030 |
| 9 | Begoniaceae | *Begonia guangxiensis* | NC_046385 |
| 10 | Begoniaceae | *Begonia gulongshanensis* | NC_063513 |
| 11 | Begoniaceae | *Begonia handelii* | NC_065245 |
| 12 | Begoniaceae | *Begonia pulchrifolia* | NC_045096 |
| 13 | Begoniaceae | *Begonia versicolor* | NC_047450 |
| 14 | Cucurbitaceae | *Benincasa hispida* | NC_056352 |
| 15 | Bixaceae | *Bixa orellana* | NC_041550 |
| 16 | Theaceae | *Camellia cuspidata* | NC_022459 |
| 17 | Theaceae | *Camellia japonica* | NC_036830 |
| 18 | Caricaceae | *Carica papaya* | NC_010323 |
| 19 | Dilleniaceae | *Dillenia indica* | NC_042740 |
| 20 | Dilleniaceae | *Dillenia turbinata* | NC_062798 |
| 21 | Frankeniaceae | *Frankenia laevis* | NC_041277 |
| 22 | Frankeniaceae | *Frankenia pulverulenta* | NC_041278 |
| 23 | Cistaceae | *Helianthemum songaricum* | NC_053644 |
| 24 | Tamaricaceae | *Myricaria paniculata* | NC_041270 |
| 25 | Passifloraceae | *Passiflora auriculata* | NC_038119 |
| 26 | Passifloraceae | *Passiflora lutea* | NC_043815 |
| 27 | Ochnaceae | *Sauvagesia rhodoleuca* | MW772237 |
| 28 | Dipterocarpaceae | *Shorea pachyphylla* | NC_040966 |
| 29 | Dipterocarpaceae | *Shorea zeylanica* | NC_040965 |
| 30 | Stachyuraceae | *Stachyurus chinensis* | NC_058889 |
| 31 | Stachyuraceae | *Stachyurus retusus* | NC_058890 |
| 32 | Tamaricaceae | *Tamarix chinensis* | NC_040943 |
| 33 | Cucurbitaceae | *Thladiantha dubia* | NC_046855 |
| 34 | Violaceae | *Viola mirabilis* | NC_041582 |
| 35 | Violaceae | *Viola philippica* | NC_052919 |
